# International Newborn Screening: Where Are We in Saudi Arabia?

**DOI:** 10.1007/s44197-024-00263-z

**Published:** 2024-06-26

**Authors:** Noara Alhusseini, Yara Almuhanna, Lama Alabduljabbar, Soaad Alamri, Maryam Altayeb, Ghadi Askar, Noor Alsaadoun, Khadijah Ateq, Mariam M. AlEissa

**Affiliations:** 1 Medical School, Alfaisal University, Riyadh, Saudi Arabia; 2 Public Health Authority, Public health Lab, Riyadh, Saudi Arabia; 3 King Abdullah International Medical Research Center, King Saud Bin Abdulaziz University for Health Science, Riyadh, Saudi Arabia

**Keywords:** Newborn screening program, Prevention, Early intervention, Noncommunicable diseases, Saudi Arabia

## Abstract

Newborn screening (NBS) programs are believed to play an important role in the decrease of infant mortality rates in many countries. This is achieved through offering early detection and treatment of many genetic as well as metabolic disorders prior to the onset of symptoms. Our paper examines NBS across seven diverse nations: Saudi Arabia, the United States, Japan, Singapore, Canada, Australia, and the United Kingdom. This paper discusses the diseases screened for by each country, latest additions, as well as future recommendations, when applicable. Employing a comparative approach, we conducted a comprehensive review of the most recent published literature on NBS programs in each country and subsequently examined their latest implemented NBS guidelines as outlined on their respective official government health sector websites. We then reviewed the economic feasibility of each of these programs and factors that affect implementation and overall benefit. While all six countries employ well-developed programs, variations are observed. Those variations are mainly attributed to disparities in access, resource scarcity, financial availability, as well as ethical and cultural considerations. From a local perspective, we recommend conducting further population-based studies to assess the epidemiological data in relation to the disease burden on the country’s economy. Moreover, we recommend updating national and international guidelines to contain a more comprehensive approach on policies, operation, and sustainability to deliver a service through the lens of value-based healthcare.

## Introduction

A screening program is a tool that allows us to detect diseases in their early stages in a target group, allowing for early treatment and lowering the rates of morbidity and mortality [1]. Screening is an essential tool in the healthcare sector used to identify people in an apparently healthy population who are at higher risk of a health condition, so that an early treatment or intervention can be offered, thereby reducing the burden of disease within the population [2]. Screening programs are implemented worldwide and serve as a preventative measure; this includes newborn screening programs (NBS) [3].

The WHO created a regulatory document in 2005 titled “The International Health Regulations (IHR).” This document provides an overarching legal framework that defines countries’ rights and obligations in handling public health events and emergencies that have the potential to cross borders [2]. The IHR is an instrument of international law that is legally binding on 196 countries, including the 194 WHO Member States, which covers screening for communicable diseases [4]. Policy-makers, health professionals, and the public often seem unaware of the potential challenges of screening, its cost and burden on the health system, and the need for strong quality assurance [4]. WHO designed a guide for policymakers and public health leaders involved in planning, designing, and implementing screening programs in the WHO European Region. It describes various aspects policymakers should consider before starting, continuing, or stopping a screening program and the operational, monitoring, and evaluation aspects of implementation. This guide forms part of WHO’s efforts to increase the effectiveness of screening programs within the Region, maximizing benefits and minimizing harm [5].

Not all countries conduct decision-making and implementation at the national level [6]. The Netherlands, New Zealand, and the UK are responsible for recommendations and national implementation of screening programs, whereas in Belgium, France, and Germany, implementation is delegated to regional and local authorities. In Australia, Canada, and Sweden, both the decision-making and implementation are made at lower levels [6]. Variable consideration also exists in each country’s approach to starting or continuing screening programs, and this includes individual differences in screening systems, selection of screening topics, criteria for assessing evidence, decision-making processes, health systems, and structures [7].

## Methodology

In this paper, a review of the international screening of seven diverse nations was conducted. A literature search was conducted on PubMed, Google Scholar, and governmental and public health organization websites for the keywords “newborn screening” “Public Health” and “newborn genetic testing”. Quality was assessed based on methodological clarity, data robustness, and source credibility, facilitating a comparative analysis of screening programs in selected nations. The authors included recent studies “if available”, peer-reviewed studies, or official governmental websites.

### International Newborn Screening Guidelines

Newborn screening is considered to be an instrument that promotes the wellbeing of a society, and it achieves the UN Sustainable Development Goals (SDGs) [7]. In particular the goals related to ending preventable deaths of newborns and children under the age of 5 years (SDG target 3.2), as well as reducing non-communicable diseases through prevention and treatment (SDG target 3.4). It also reduces societal and family financial burdens thereby impacting SDG 1 by ending poverty. It promotes equality by ensuring equitable access to screening regardless of gender or socioeconomic status thereby reducing inequalities by ensuring universal screening for all newborns (SGD 10) [8].

Table 1 summarizes the core diseases detected by newborn screening programs established by seven different countries, including Saudi Arabia, the United States, Japan, Singapore, Canada, Australia, and the United Kingdom. The wide range of disorders covered by each country demonstrates that each individual screening program is based on the epidemiological, financial, and economic resources of that country [9]. Taking the United States as an example, their evaluation criteria for selected conditions was composed of three aspects; clinical characteristics, analytical characteristics, and future diagnosis and management plans of the condition. A scoring and ranking system is then developed and compared for the selection criteria [10].

**Table 1:** Newborn screening test in different countries compared to Saudi Arabia

	Saudi Arabia [8]	United States* [10]	Japan† [11]	Singapore* [12]	Canada [7]	Australia [13]	United Kingdom [14]
3-Hydroxy-3-methylglutaric aciduria	✔	✔	✔	✔		✔	
3-Methylcrotonyl-CoA carboxylase deficiency	✔	✔	✔				
ß-Ketothiolase deficiency	✔	✔		✔		✔	
Glutaric acidemia type I	✔	✔	✔	✔	✔	✔	✔
Glutaric Acidemia Type II multiple acyl-CoA-dehydrogenase deficiency	✔					✔	
Holocarboxylase synthase deficiency		✔	✔			✔	
Isovaleric acidemia	✔	✔	✔	✔	✔	✔	✔
Methylmalonic acidemia (cobalamin disorders)	✔	✔	✔	✔ **	✔	✔	
Methylmalonic acidemia (methylmalonyl-CoA mutase)		✔		✔	✔	✔	
Propionic acidemia	✔	✔	✔	✔	✔	✔	
Carnitine uptake defect/carnitine transport defect		✔		✔	✔	✔	
Long-chain L-3 hydroxyacyl-CoA dehydrogenase deficiency		✔		✔	✔	✔	
Medium-chain acyl-CoA dehydrogenase deficiency	✔	✔	✔	✔	✔	✔	✔
Trifunctional protein deficiency		✔	✔	✔	✔	✔	
Very long-chain acyl-CoA dehydrogenase deficiency	✔	✔	✔	✔	✔	✔	
Argininosuccinic aciduria	✔	✔	✔	✔	✔	✔	
Citrullinemia, type I	✔	✔	✔	✔	✔	✔	
Classic phenylketonuria	✔	✔	✔	✔	✔	✔	✔
Homocystinuria	✔	✔	✔	✔	✔	✔	✔
Maple syrup urine disease	✔	✔	✔	✔	✔	✔	✔
Tyrosinemia, type I	✔	✔		✔	✔		✔
Congenital adrenal hyperplasia	✔	✔	✔		✔	✔††	
Primary congenital hypothyroidism	✔	✔	✔	✔	✔	✔	✔
Biliary atresia					✔		
βeta-thalassemia	✔						
S, βeta-thalassemia		✔			✔		
S, C disease		✔			✔		
S, S disease (Sickle cell anemia)	✔	✔			✔		✔
Biotinidase deficiency	✔	✔			✔		
Classic galactosemia	✔	✔	✔		✔		
Congenital heart disease		✔			✔		✔
Cystic fibrosis		✔			✔	✔	✔
Glycogen Storage Disease Type II (Pompe)		✔					
Guanidinoacetate Methyltransferase Deficiency		✔			✔		
Hearing loss		✔		✔			✔
Mucopolysaccharidosis Type I		✔			✔		
Mucopolysaccharidosis Type II		✔					
Severe combined Immunodeficiencies		✔			✔		
Spinal Muscular Atrophy		✔	✔		✔		
X-linked Adrenoleukodystrophy		✔					
Tyrosinemia, type II and III						✔	✔
Carnitine palmitoyltransferase I deficiency	✔		✔			✔	
Carnitine palmitoyltransferase II deficiency			✔			✔	
Carnitine acylcarnitine translocase deficiency						✔	
G6PD	✔			✔			
Congenital cataracts							✔
Methylmalonic Acidemia Cobalamin defects C, D v2						✔	

* Core diseases are included only in this table; secondary diseases are not included

** Singapore deals with MMA and cobalamin A&B as two sperate tests, whereas all other countries consider them one

**†** Lysosomal diseases, including Gaucher, Fabry’s, Pompe disease, and mucopolysaccharidosis type 1 and 2 are only available in some areas of Japan

†† 21 hydroxylase deficiency only

Saudi Arabia’s NBS was implemented in 2005 nationwide as a mandatory measure, and currently includes 18 disorders according to the official Ministry of Health (MoH) website, with the inclusion of hemoglobinopathies underway [11]. As for the United States, each state has its own guidelines and screening programs that are not mandated but instead adapted as an opt-out policy [11]. However, there is a Recommended Uniform Screening Panel (RUSP) by the Secretary of the Department of Health and Human Services, which is included in Table 1 [11]. As of January 2023, the RUSP panel lists 38 core conditions of which screening for is highly recommended, and 26 secondary conditions of which screening for is optional [11]. It should be noted that due to the scope of this paper secondary diseases are not included in Table 1; this paper covers only what is actively recommended and implemented. In the case of Japan, their mass screening project was first started in 1977, which initially included only 6 diseases. However, after the introduction of tandem mass spectrometers, screening for 20 diseases is now officially implemented nationwide according to the official Japanese Society for Neonatal Screening. Latest additions are lysosomal disorders which are being studied in many areas in Japan and are soon to be implemented nationwide, but not added in this table again for it has not been officiated yet [12]. Provides an in-depth look into Japan’s newborn screening (NBS) program, focusing on its evolution, technological advancements like tandem mass spectrometry, and the critical role of quality assurance and standardization [7]. As for Australia, according to the Australian Government Department of Health and Aged Care, 27 diseases have been approved and implemented for screening by the health minister as of June 2023 [13]. On the other hand, in Canada, according to Newborn Screening Ontario, 30 diseases have been approved to be part of the newborn screening program [14]. Shifting to Singapore, according to an article published on July 2021 in The Singapore Medical Journal, Singapore screens for 20 primary diseases, and additionally 19 secondary diseases [15]. The secondary diseases are not included in this table for the same reason discussed above [16]. Lastly, for the United Kingdom, each of its four countries, Wales, England, Northern Ireland, and Scotland sets its own screening program based upon the official recommendations by the UK National Screening Committee (UK NSC) [17]. The diseases recommended by the UK NSC only are included in Table 1.

### Economic Feasibility of Newborn Screening Programs

Key factors that come into play with the implementation of newborn screening programs worldwide are the consideration of their economic feasibility and overall benefit in comparison [18]. The priority of newborn screening implementation varies from high to middle-low income countries due to competition with other health considerations, such as infectious disease control, immunization, and malnutrition. Economic determinants such as limited access to resources, poor economies, governmental instability, as well as local culture and geographic location influence government prioritization, public acceptance, and health practitioner cooperation/involvement of such programs [19].

For these reasons, despite technological development and worldwide accessibility to screening programs, many countries have yet to adapt them. According to Zeybek, the known percentage of newborns screened is 100% in the United States, 78% in Europe, 32% in Latin America, 26% in the Middle East and North Africa, 13% in Asia-Pacific, and 0% in Central Africa [20]. As discussed previously, NBS is a national program in countries such as Saudi Arabia and the United Kingdom, but in federal governments such as the United States and Canada, state, provincial, or regional governments carry out and decide which conditions should be screened under public health authority [21]. In federally governed populations with access to NBS programs, an opt-out policy can be implemented, requiring testing for all newborns unless parents or guardians decline to do so for religious factors or otherwise [22].

Economic evaluations of health interventions generally fall into 2 categories, cost-effectiveness analyses (CEAs) and cost-benefit analyses (CBAs). Cost-effectiveness analyses (CEAs) estimate the net costs of interventions and the numbers of outcomes achieved, whereas cost‐benefit analyses (CBAs) express costs of both healthy and nonhealthy outcomes in common monetary values, reporting estimates of net benefit in absolute terms as well as relative benefit‐cost ratios [21]. In the context of newborn screening, a key comparison is that of costs as opposed to quality-adjusted life-year (QALY), measured by CEAs [23]. QALY helps establish the effects of health interventions on mortality and morbidity into a single index; it mathematically measures health outcomes where 0 indicates death and 1 indicates full health [24]. Furthermore, a measure of incremental change in costs divided by the incremental change in health outcome, known as incremental cost-effectiveness ratio (ICER), helps provide the cost per life year gained [24]. These measures are essential in the determination of cost-effective NBS programs worldwide.

Technologies introduced in the 1990s such as electrospray ionization (ESI) and tandem mass spectrometry (MS/MS) have allowed for the detection of multiple diseases from a single blood spot (‘heel prick’), providing relatively cheap access to allow for screening, and have thus become routine [25]. This primarily allows for cost-effective detection of inborn errors of metabolism. Isoelectric focusing (IEF) is another common method that is used to detect hemoglobinopathies in half of all labs participating in the CDC Newborn Screening Quality Assurance Program’s proficiency testing report [26]. Other relatively newer techniques, such as next-generation sequencing (NGS) technology provide a second-tier for some conditions to detect underlying genetic mutations in disorders such as cystic fibrosis (CF) [27].

The cost of these technologies per newborn is influenced directly by the number of diseases screened for in each country, and can be increased by various factors such as accreditation costs, maintenance, quality control measures, and specialists [25–28]. For example, in Europe, the reported cost of screening per newborn using NGS was estimated to be approximately €1 in Moldova where 2 conditions are screened for, as opposed to €43.24 in the Netherlands where 17 conditions are screened for. Tandem mass spectrometry is determined to be generally cost-effective in countries such as Canada, Australia, the United States, and Japan [29–32], however, some countries such as Thailand have found neonatal screening using this technique to be cost-ineffective due to limited resources [33].

In spite of these cost constraints, a recent review of newborn screening programs worldwide conducted by Therrell et al., found that NBS programs become more crucial as a public health prevention measure as the infant mortality rate (IMR) approaches 20 deaths per thousand. As the IMR nears single digits, many countries have the capacity to establish a NBS system targeting one or multiple disorders [34].

### Ethics of Newborn Screening

Furthermore, individual awareness and autonomy regarding NBS has various ethical implications that warrant careful consideration. One concern is the balance between beneficence and autonomy, as screening carries the probability of false positives, leading to psychological distress for families and individuals. Moreover, the subsequent burden of unnecessary medical interventions in this case raises questions about the appropriate scope and accuracy of screening protocols [35]. Additionally, issues related to consent, privacy, and the potential for discrimination based on genetic information underscore the need for robust ethical frameworks to guide the implementation and governance of newborn screening programs. Though these protocols differ from country to country, with some considering screening mandatory and others applying an opt-out model instead, addressing these ethical dilemmas is imperative to ensure that the benefits of early detection are maximized while respecting the rights and dignity of newborns and their families [35].

### Limitations

There are many limitations of this study. First, selection bias due to the selection of the available studies within the chosen search engines and keyword selection. Second, publication bias because usually certain studies may not pass publication criteria. Third, data availability. Some relevant studies might be inaccessible due to language barriers, subscription requirements, or limited access to certain databases.

## Conclusion

Newborn screening (NBS) programs play an important role in decreasing infant mortality rates in many countries. In our paper, we highlighted the differences between international newborn screening programs and national newborn screening programs in Saudi Arabia, and discussed their economic feasibility, which involves many crucial variables that affect implementation and overall benefit.

Despite the evidence supporting the importance of implementing newborn screening programs, high variability exists worldwide and no global consensus has been met. Creating screening programs is a complex and multifaceted process that depends on intrinsic factors specific to each country. Though some countries have high disease incidence, legislative and economic factors limit the administration of preventative screening. As such, not all countries mandate said programs, which influences the extent to which they are effective. Moreover, some considerable economic determinants include poor economies, lack of resources, governmental instability, geographic location, and local culture.

Nationally, as per the Ministry of Health, the Kingdom of Saudi Arabia includes 18 diseases that are part of its NBS program with the addition of hemoglobinopathies underway. In comparison, the United States follows a NBS program that screens for 38 core conditions, Japan’s NBS program screens for 20 conditions, Australia screens for 27 diseases, Canada screens for 30 diseases, and 20 primary diseases are screened for in Singapore’s NBS program. Lastly, the United Kingdom’s NBS program offers different guidelines and programs for each of the different regions within the UK as recommended by the UK National Screening Committee (UK NSC).

With this in mind, there remains a gap in the available data regarding screening programs and their utilization in various countries worldwide. From a local perspective, we recommend conducting further population-based studies to assess the epidemiological data in Saudi Arabia in relation to the disease burden on the country’s economy. This is essential particularly due to the high rate of consanguinity within the population and consequently higher prevalence of genetic diseases. Key measures to be considered include the incremental cost-effectiveness ratio (ICER) that aids in providing the cost for each life year gained, and is essential in establishing NBS programs that are cost-effective. Moreover, we recommend updating national and international guidelines to contain a more comprehensive approach on policies, operation, and sustainability to deliver a service through the lens of value-based healthcare.

## Data Availability

No datasets were generated or analysed during the current study.

## Abbreviations

NBS: Newborn screening
ICER: Incremental cost-effectiveness ratio
UK NSC: UK National Screening Committee
NGS: Next-generation sequencing
CF: Cystic fibrosis
IEF: Isoelectric focusing
RUSP: Recommended Uniform Screening Panel
MoH: Ministry of Health
IHR: International Health Regulations
WHO: World Health Organazation
